# Comparative exposure and risk assessment of heavy metals, nutrients, and organochlorine pesticides in cow and plant-based milks

**DOI:** 10.1038/s41598-026-44766-0

**Published:** 2026-04-09

**Authors:** Sonya Good, Chukwunonso Anakwue, Tuan Phan

**Affiliations:** Department of Chemistry, Texas Southern University, Houston, TX United States of America

**Keywords:** Heavy metals, Organochlorine pesticides, Plant-based milk, Cow milk, Environmental contamination, Food safety, Environmental sciences, Risk factors

## Abstract

Cow milk and plant-based milk alternatives (PBMAs) are widely consumed beverages, yet both can act as pathways for exposure to environmental contaminants originating from soil, water, and legacy agricultural practices. In this study, we quantified heavy metals (lead (Pb), cadmium (Cd), chromium (Cr), and arsenic (As)), macro- and micronutrients, and 24 organochlorine pesticides (OCPs) in cow milk and seven PBMAs (almond, soy, oat, coconut, hemp, rice, and cashew). Twenty-two commercial products were analyzed using inductively coupled plasma mass spectrometry (ICP-MS) and gas chromatography with electron-capture detection (GC-ECD). PBMAs exhibited higher and more variable concentrations of Cr, As, and Cd than cow milk, with rice and hemp milks showing the highest arsenic and chromium levels, respectively. Lead concentrations were comparable across milk types. Fortified PBMAs showed elevated calcium (Ca), sodium (Na), and magnesium (Mg) relative to cow milk, reflecting formulation-driven rather than intrinsic nutritional differences. All milk categories contained detectable residues of multiple OCPs, including dicofol, mirex, and hexachlorobenzene, highlighting the persistence of legacy pesticides in modern food products. Although concentrations generally remained below regulatory limits, the widespread occurrence of both metals and OCPs underscores the need for continued monitoring and improved transparency for both dairy and plant-based milks.

## Introduction

Milk is a globally consumed dietary staple and an important source of essential mineral nutrients such as calcium (Ca), magnesium (Mg), sodium (Na), potassium (K), iron (Fe), copper (Cu), zinc (Zn), and manganese (Mn). These nutrients support bone development, neuronal signaling, electrolyte balance, and numerous metabolic processes^1–6^. Cow milk is widely recognized for its bioavailable Ca content and its contribution to growth and overall health. However, several factors, including lactose intolerance, dairy allergies, health preferences, cultural dietary patterns, and perceived digestibility, have contributed to the increasing popularity of plant-based milk alternatives (PBMAs)^7–11^.

PBMAs vary widely in mineral composition due to natural differences in plant materials and the common practice of supplementation or fortification, wherein manufacturers add Ca, Mg, Na, or other minerals to enhance nutritional profiles^12^. Both diary and PBMA products may also contain contaminants such as heavy metals and pesticide residues originating from soil, irrigation water, atmospheric deposition, and legacy agricultural practices. Heavy metals, including lead (Pb), cadmium (Cd), chromium (Cr), and arsenic (As), pose significant risks due to their bioaccumulation potential and associations with neurotoxicity, kidney damage, and carcinogenicity^13–18^.

Organochlorine pesticides (OCPs), despite widespread bans, remain persistent in soils and sediments and can enter food products through environmental pathways^19–26^.

Although some studies have begun to examine contaminants in PBMAs, the number of such investigations remains small compared to the extensive literature on cow’s milk. This represents a research gap given the growing consumption of PBMAs. Because PBMAs are derived from diverse biological matrices such as nuts, grains, seeds, and legumes, their contaminant uptake patterns may be markedly different from those of dairy milk^27^. For example, rice is known to readily accumulate heavy metals from soil and irrigation water^20,28,29^, and hemp demonstrates strong uptake of Cr and Pb under certain agricultural conditions^30,31^.

This study aimed to address existing gaps in the literature by conducting a comprehensive comparative analysis of heavy metals, essential mineral nutrients, and OCP residues in both cow milk and diverse PBMAs. The primary objective was to quantify concentrations of heaving metals using inductively coupled plasma mass spectrometry (ICP-MS), while simultaneously measuring key macro- and micro-mineral nutrients to evaluate nutritional variability across products. Additionally, the study sought to detect legacy OCPs through gas chromatography with an electron capture detector (GC-ECD), recognizing that these persistent contaminants may remain detectable in food products regardless of geographical origin.

Based on the compositional differences between dairy milk and PBMAs, we hypothesized that PBMAs would exhibit greater variability in mineral nutrient levels due to differences in plant matrices and common fortification practices. We further expected that PBMAs derived from crops known for high metal uptake - such as rice, hemp, and soy - might display higher concentrations of certain heavy metals compared to cow milk. Finally, given the environmental persistence of OCPs, we anticipated detectable residues across all milk categories, independent of sourcing, reflecting long-range environmental transport and historical agricultural applications rather than contemporary regional farming practices.

## Materials and methods

### Sample selection and classification

A total of twenty-two commercially available milk products were purchased from major grocery retailers in Houston, Texas, USA. These products included cow milk and seven categories of PBMAs: almond, soy, oat, coconut, hemp, rice, and cashew. Samples were selected based on market availability and consumer prevalence rather than geographic sourcing, as commercial PBMAs often rely on nationally or internationally sourced raw ingredients^32,33^. The study encompassed four brands of cow’s milk and almond milk, three brands of soy and coconut milks, and two brands of oat, rice, hemp, and cashew milks. All samples were in liquid form and packaged in their original retail containers, which included high-density polyethylene (HDPE), aseptic cartons, and glass bottles. Container material was recorded to evaluate potential influence on analyte concentrations; no statistically meaningful differences associated with packaging were observed.

### Sample storage and handling

Immediately after purchase, samples were transported to the laboratory under temperature-controlled conditions and stored at 4 °C in the dark to minimize degradation and photochemical changes. All analyses were performed within 72 h of purchase to limit variability associated with storage duration, following established sample-handling practices^34^.

### ICP-MS analysis for heavy metals and mineral nutrients

#### Sample preparation and digestion

Milk samples were prepared for ICP-MS analysis using microwave-assisted acid digestion, following established protocols for dairy milk and plant-based milk alternatives^35,36^. For each sample, 4 mL of well-homogenized milk was transferred into a Teflon digestion vessel, followed by the addition of 10 mL of 70% nitric acid (HNO₃). Microwave digestion was performed at 200 °C and 800 psi for 15 min. After cooling, each digestate was diluted to a final acid concentration of 2% HNO₃ using ultrapure deionized water.

#### Instrumentation and operating conditions

Heavy metals (Cr, As, Pb, Cd) and macro- and micro-mineral nutrients (Ca, Mg, Na, K, Fe, Cu, Mn) were quantified using an Agilent 7900 ICP-MS (USA). Calibration standards (0.05–100 µg L⁻¹) were prepared using multi-element stock solutions. Helium collision gas mode was employed to minimize polyatomic interferences. Internal standards (scandium, yttrium, terbium) were introduced online to correct for matrix effects and instrumental drifts^37–43^ need to add reference #31 here.

### GC-ECD analysis for organochlorine pesticides

#### Extraction and cleanup

OCP extraction followed the USEPA 8081B protocol using liquid-liquid extraction with a 1:1 hexane-acetone mixture^44^. The organic layer was subjected to silica gel cleanup to remove lipids and co-extracted matrix components^45^.

#### Instrumental analysis

An Agilent 6890 GC-ECD (USA) and dual Restek Rtx-CL Pesticides columns (Restek, USA) was used for pesticide detection. GC conditions employed nitrogen as the carrier gas (4.3 mL/min), with the injector and ECD set at 250 °C and 340 °C, respectively. The oven program initiated at 120 °C (3-min hold) and ramped to 310 °C (1-min hold). Calibration used EPA 500-series OCP standards spanning 0.5–500 µg/kg.

### Quality control

Instrument precision was assessed through triplicate injections of each sample. Matrix spike samples were prepared by fortifying representative milk matrices with known analyte concentrations, and recoveries of 80–120% were accepted in accordance with AOAC method-validation guidelines^46^.

### Statistical analysis

All concentrations are reported as mean ± standard deviation (SD) across brands for each milk type. Each commercial brand was analyzed in triplicate, and the arithmetic mean of triplicate measurements was used to represent each brand. Brand-level means were treated as independent observations for summary statistics. The number of brands analyzed per milk type was four for cow, almond, and soy milks; three for coconut milk; and two for oat, rice, hemp, and cashew milks. Accordingly, SD reflects between-brand variability rather than analytical precision. Given the exploratory nature of the study and the limited number of brands per milk type, formal hypothesis testing was not applied unless otherwise noted.

## Results

### Heavy metal concentrations

Measured concentrations of Cr, As, Cd, and Pb in cow milk and PBMAs are summarized in Table 1. Detectable levels of heavy metals were predominantly observed in PBMAs, whereas cow milk consistently exhibited the lowest concentrations across most analytes.

Chromium concentrations ranged from 0.11 to 0.68 µg L⁻¹ across all samples, with cow milk exhibiting the lowest concentration (0.11 µg L⁻¹). Among PBMAs, hemp milk contained the highest Cr concentration (0.68 µg L⁻¹), followed by oat, almond, and cashew milks. Soy, coconut, and rice milks exhibited comparatively lower Cr concentrations, ranging from 0.23 to 0.26 µg L⁻¹.

**Table 1:** Heavy metal concentrations in cow and plant-based milk alternatives (µg L⁻¹).^1^.

Milk type	Cr	As	Cd	Pb
Cow	0.11 ± 0.06	0.02 ± 0.01	0.03 ± 0.01	0.20 ± 0.10
Almond	0.43 ± 0.63	0.07 ± 0.05	0.04 ± 0.04	0.18 ± 0.07
Coconut	0.24 ± 0.10	0.13 ± 0.04	0.05 ± 0.02	0.28 ± 0.23
Soy	0.23 ± 0.05	0.08 ± 0.02	0.17 ± 0.03	0.28 ± 0.06
Oat	0.49 ± 0.09	0.13 ± 0.03	0.10 ± 0.04	0.26 ± 0.06
Rice	0.26 ± 0.05	0.64 ± 0.27	0.04 ± 0.01	0.26 ± 0.06
Hemp	0.68 ± 0.55	0.28 ± 0.22	0.05 ± 0.02	0.21 ± 0.03
Cashew	0.41 ± 0.05	0.05 ± 0.02	0.03 ± 0.02	0.18 ± 0.02

^1^Values are reported as mean ± SD across brands (*n* = 2–4). Each brand was analyzed in triplicate, and brand-level means were used to calculate milk-type means and SD.

Arsenic concentrations ranged from 0.02 to 0.64 µg L⁻¹, with cow milk again showing the lowest concentration. Among PBMAs, cashew, almond, and soy milks contained 0.05, 0.07, and 0.08 µg L⁻¹ of As, respectively. The highest As concentration was observed in rice milk (0.64 µg L⁻¹), followed by hemp milk (0.28 µg L⁻¹), while coconut and oat milks exhibited intermediate concentrations of 0.13 µg L⁻¹. Cadmium concentrations ranged from 0.03 to 0.17 µg L⁻¹ across samples. With the exception of soy milk (0.17 µg L⁻¹) and oat milk (0.10 µg L⁻¹), all milk samples contained Cd concentrations between 0.03 and 0.05 µg L⁻¹. Lead concentrations ranged from 0.18 to 0.28 µg L⁻¹ across all samples. Unlike the other metals, Pb concentrations in cow milk were comparable to those measured in PBMAs.

### Nutrient composition

#### Macronutrients

Measured concentrations of Na, Mg, K, and Ca in cow milk and PBMAs are summarized in Table 2. Substantial variability in macronutrient concentrations was observed across milk types.

**Table 2:** Macronutrient concentrations in cow and plant-based milk alternatives (µg L⁻¹).^1^.

Milk type	Na	Mg	K	Ca
Cow	14,919 ± 4 712	2,593 ± 263	64,643 ± 22 340	18,735 ± 7 543
Almond	16,250 ± 10 210	610 ± 344	25,200 ± 14 570	20,700 ± 12 190
Coconut	3,400 ± 2 140	670 ± 420	11,500 ± 7 820	9,800 ± 6 520
Soy	6,250 ± 2,480	1,870 ± 130	39,000 ± 7,910	7,800 ± 3,610
Oat	9,300 ± 1,140	450 ± 55	56,000 ± 9,280	11,500 ± 3,840
Rice	9,900 ± 1,010	220 ± 32	2,800 ± 460	5,600 ± 1,710
Hemp	11,050 ± 1,200	2,100 ± 1,470	9,800 ± 7,460	6,200 ± 5,260
Cashew	6,600 ± 4,780	860 ± 52	7,300 ± 470	1,100 ± 420

^1^Values are reported as mean ± SD across brands (*n* = 2–4). Each brand was analyzed in triplicate, and brand-level means were used to calculate milk-type means and SD.

Sodium concentrations ranged from 3,400 to 16,250 µg L⁻¹ across samples. Almond milk exhibited the highest Na concentration (16,250 µg L⁻¹), followed by cow milk (14,919 µg L⁻¹). Coconut and soy milks exhibited the lowest Na concentrations. Magnesium concentrations ranged from 220 to 2,593 µg L⁻¹ across samples. Cow milk exhibited the highest Mg concentration (2,593 µg L⁻¹). Among PBMAs, hemp milk (2,100 µg L⁻¹) and soy milk (1,870 µg L⁻¹) contained comparatively higher Mg concentrations, while the remaining PBMAs ranged from 220 to 860 µg L⁻¹. Potassium concentrations ranged from 2,800 to 64,643 µg L⁻¹ across samples. Cow milk exhibited the highest K concentration (64,643 µg L⁻¹). Among PBMAs, oat milk (56,000 µg L⁻¹) and soy milk (39,000 µg L⁻¹) contained elevated K concentrations, whereas coconut, hemp, rice, and cashew milks exhibited lower concentrations ranging from 2,800 to 11,500 µg L⁻¹.

Calcium concentrations ranged from 1,100 to 20,700 µg L⁻¹ across samples. Cow milk contained 18,735 µg L⁻¹ of Ca. Among PBMAs, almond milk exhibited the highest Ca concentration (20,700 µg L⁻¹), followed by oat milk (11,500 µg L⁻¹). Soy, rice, hemp, and cashew milks contained lower Ca concentrations, ranging from 1,100 to 7,800 µg L⁻¹.

#### Micronutrients

Measured concentrations of iron (Fe), copper (Cu), zinc (Zn), and manganese (Mn) in cow milk and PBMAs are summarized in Table 3. Substantial variability in micronutrient concentrations was observed across milk types.

Iron concentrations ranged from 21 to 114 µg L⁻¹ across samples. Cow milk contained 21 µg L⁻¹ of Fe. Among PBMAs, soy milk exhibited the highest Fe concentration (114 µg L⁻¹), followed by hemp milk (98 µg L⁻¹). Almond, coconut, oat, rice, and cashew milks contained intermediate Fe concentrations ranging from 29 to 59 µg L⁻¹.

Copper concentrations ranged from 23 to 65 µg L⁻¹ across samples. Cow milk contained 63 µg L⁻¹ of Cu. Among PBMAs, soy milk exhibited the highest Cu concentration (65 µg L⁻¹), while coconut milk contained the lowest concentration (23 µg L⁻¹). The remaining PBMAs exhibited Cu concentrations ranging from 27 to 38 µg L⁻¹.

Zinc concentrations ranged from 17 to 203 µg L⁻¹ across samples. Cow milk exhibited the highest Zn concentration (203 µg L⁻¹). Among PBMAs, soy milk (74 µg L⁻¹) and hemp milk (63 µg L⁻¹) contained comparatively higher Zn concentrations, whereas almond, coconut, oat, rice, and cashew milks ranged from 17 to 46 µg L⁻¹.

Manganese concentrations ranged from 1.2 to 47 µg L⁻¹ across samples. Cow milk exhibited the lowest Mn concentration (1.2 µg L⁻¹). Among PBMAs, soy milk exhibited the highest Mn concentration (47 µg L⁻¹), followed by hemp (39 µg L⁻¹) and oat milks (26 µg L⁻¹). Almond, coconut, rice, and cashew milks contained Mn concentrations ranging from 9.3 to 16 µg L⁻¹.

**Table 3:** Micronutrient concentrations in cow and plant-based milk alternatives (µg L⁻¹).^1^.

Milk type	Fe	Cu	Zn	Mn
Cow	21 ± 10	63 ± 28	203 ± 65	1.2 ± 0.4
Almond	43 ± 26	33 ± 11	20 ± 9	9.6 ± 4.5
Coconut	59 ± 36	23 ± 9	36 ± 25	9.3 ± 2.1
Soy	114 ± 18	65 ± 11	74 ± 6	47 ± 6
Oat	44 ± 7	38 ± 4	32 ± 8	26 ± 7
Rice	29 ± 23	27 ± 3	17 ± 4	10 ± 4
Hemp	98 ± 68	30 ± 16	63 ± 61	39 ± 42
Cashew	57 ± 5	37 ± 14	46 ± 4	16 ± 2

^1^Values are reported as mean ± SD across brands (*n* = 2–4). Each brand was analyzed in triplicate, and brand-level means were used to calculate milk-type means and SD.

### Organochlorine pesticide residues concentrations

Measured concentrations of 24 OCPs determined by GC–ECD in cow milk and PBMAs are summarized in Table 4. Detectable residues of all target OCPs were observed across all milk categories, with substantial variability among milk types. Overall OCP concentrations ranged from low µg L⁻¹ levels to several hundred µg L⁻¹ depending on compound and milk type. Hexachlorobenzene (HCB), mirex, kepone, toxaphene, and dicofol were among the most abundant compounds detected across samples.

Dicofol exhibited the highest concentrations among all OCPs, ranging from 96.2 to 948.4 µg L⁻¹. Cow milk contained 945.6 µg L⁻¹ of dicofol. Among PBMAs, soy milk exhibited the highest dicofol concentration (948.4 µg L⁻¹), while coconut and hemp milks contained substantially lower concentrations (96.2 µg L⁻¹).

**Table 4:** Organochlorine pesticide (OCP) concentrations in cow and plant-based milk alternatives (µg L⁻¹).^1^.

OCP	Cow	Almond	Coconut	Soy	Oat	Rice	Hemp	Cashew
α-Chlordane	35.4 ± 1.0	35.9 ± 1.3	3.68 ± 0.05	35.6 ± 0.2	35.6 ± 0.21	19.6 ± 22.5	3.68 ± 0.05	35.7 ± 1.2
γ-Chlordane	25.5 ± 0.8	25.6 ± 1.0	2.65 ± 0.04	25.6 ± 0.2	25.6 ± 0.14	129.3 ± 179.2	2.65 ± 0.04	25.7 ± 0.9
4,4-DDD	36.9 ± 1.1	37.0 ± 1.2	3.83 ± 0.05	37.0 ± 0.2	37.0 ± 0.21	20.4 ± 23.5	3.83 ± 0.05	36.9 ± 1.1
4,4-DDE	51.1 ± 1.5	51.2 ± 1.6	5.30 ± 0.08	51.2 ± 0.3	51.2 ± 0.35	28.2 ± 32.5	5.30 ± 0.08	51.0 ± 1.4
4,4-DDT	68.1 ± 2.0	68.3 ± 2.1	7.06 ± 0.10	68.3 ± 0.5	68.3 ± 0.49	37.6 ± 43.3	7.06 ± 0.10	68.2 ± 1.9
α-BHC	14.2 ± 0.4	14.3 ± 0.5	1.46 ± 0.04	14.3 ± 0.1	14.3 ± 0.07	7.8 ± 9.0	1.46 ± 0.04	14.2 ± 0.4
β-BHC	46.8 ± 1.3	46.9 ± 1.4	4.84 ± 0.07	46.9 ± 0.3	46.9 ± 0.28	25.9 ± 29.8	4.84 ± 0.07	46.8 ± 1.2
γ-BHC	21.3 ± 0.6	21.4 ± 0.6	2.21 ± 0.03	21.3 ± 0.1	21.3 ± 0.14	11.7 ± 13.5	2.21 ± 0.03	21.4 ± 0.6
Aldrin	28.4 ± 0.8	28.7 ± 0.9	2.94 ± 0.04	28.7 ± 0.1	28.7 ± 0.14	15.7 ± 18.0	2.94 ± 0.04	28.4 ± 0.8
Dieldrin	35.4 ± 1.0	35.6 ± 1.1	3.65 ± 0.05	35.6 ± 0.2	35.6 ± 0.21	19.6 ± 22.5	3.65 ± 0.05	35.4 ± 1.0
Endosulfan I	48.2 ± 1.4	48.4 ± 1.5	4.98 ± 0.07	48.4 ± 0.3	48.4 ± 0.35	26.6 ± 30.7	4.98 ± 0.07	48.2 ± 1.4
Endosulfan II	39.7 ± 1.2	39.8 ± 1.3	4.10 ± 0.06	39.8 ± 0.3	39.8 ± 0.28	21.9 ± 25.3	4.10 ± 0.06	39.7 ± 1.2
Endosulfan sulfate	35.4 ± 1.0	35.6 ± 1.1	3.65 ± 0.05	35.6 ± 0.2	35.6 ± 0.21	19.6 ± 22.5	3.65 ± 0.05	35.4 ± 1.0
Endrin	55.3 ± 1.6	55.5 ± 1.7	5.71 ± 0.08	55.5 ± 0.4	55.5 ± 0.35	30.6 ± 35.2	5.71 ± 0.08	55.3 ± 1.6
Endrin aldehyde	58.2 ± 1.7	58.3 ± 1.8	6.01 ± 0.09	58.3 ± 0.4	58.3 ± 0.42	32.1 ± 37.0	6.01 ± 0.09	58.2 ± 1.7
Endrin ketone	46.8 ± 1.3	46.9 ± 1.4	4.84 ± 0.07	46.9 ± 0.3	46.9 ± 0.28	25.9 ± 29.8	4.84 ± 0.07	46.8 ± 1.3
Heptachlor	46.8 ± 1.3	46.9 ± 1.4	4.84 ± 0.07	46.9 ± 0.3	46.9 ± 0.28	25.9 ± 29.8	4.84 ± 0.07	46.8 ± 1.3
Heptachlor epoxide	36.9 ± 1.1	37.0 ± 1.2	3.82 ± 0.05	37.0 ± 0.2	37.0 ± 0.21	20.4 ± 23.5	3.82 ± 0.05	36.9 ± 1.1
Methoxychlor	80.8 ± 2.3	81.1 ± 2.4	8.35 ± 0.12	81.1 ± 0.5	81.1 ± 0.49	44.7 ± 51.4	8.35 ± 0.12	80.8 ± 2.3
Mirex	344.5 ± 9.9	345.5 ± 10.2	35.1 ± 0.5	345.5 ± 2.1	345.5 ± 2.12	190.2 ± 218.9	35.1 ± 0.5	344.5 ± 9.9
Toxaphene	237.0 ± 6.8	237.5 ± 7.1	24.2 ± 0.3	237.5 ± 2.1	237.5 ± 2.12	130.7 ± 150.4	24.2 ± 0.3	237.0 ± 6.8
Dicofol	945.6 ± 27.4	948.4 ± 28.6	96.2 ± 1.4	948.4 ± 6.8	948.4 ± 6.0	522.7 ± 601.9	96.2 ± 1.4	945.6 ± 27.4
Hexachlorobenzene	371.5 ± 10.6	378.0 ± 11.1	38.1 ± 0.6	378.0 ± 8.2	378.0 ± 8.2	205.6 ± 236.8	38.1 ± 0.6	371.5 ± 10.6
Kepone	334.5 ± 9.9	340.5 ± 10.3	34.1 ± 0.5	340.5 ± 7.7	340.5 ± 7.7	185.2 ± 213.3	34.1 ± 0.5	334.5 ± 9.9

^1^Values are reported as mean ± SD across brands (*n* = 2–4). Each brand was analyzed in triplicate, and brand-level means were used to calculate milk-type means and SD.

Mirex concentrations ranged from 35.1 to 345.5 µg L⁻¹ across samples. Cow milk contained 344.5 µg L⁻¹ of mirex. Among PBMAs, almond and soy milks exhibited similarly elevated concentrations (345.5 and 345.5 µg L⁻¹, respectively), whereas coconut and hemp milks contained the lowest concentrations (35.1 µg L⁻¹).

Hexachlorobenzene concentrations ranged from 38.1 to 378.0 µg L⁻¹. Cow milk contained 371.5 µg L⁻¹ of HCB. Among PBMAs, almond, soy, and oat milks exhibited the highest HCB concentrations (378.0 to 378.0 µg L⁻¹), while coconut and hemp milks exhibited substantially lower concentrations.

Similar distribution patterns were observed for other OCPs, including toxaphene, kepone, and DDT-related compounds, with higher concentrations generally observed in cow, almond, soy, and oat milks, and lower concentrations in coconut and hemp milks. Rice milk exhibited greater variability across compounds, reflected by comparatively larger standard deviations.

## Discussion

### Heavy metal concentration

**Fig. 1:**
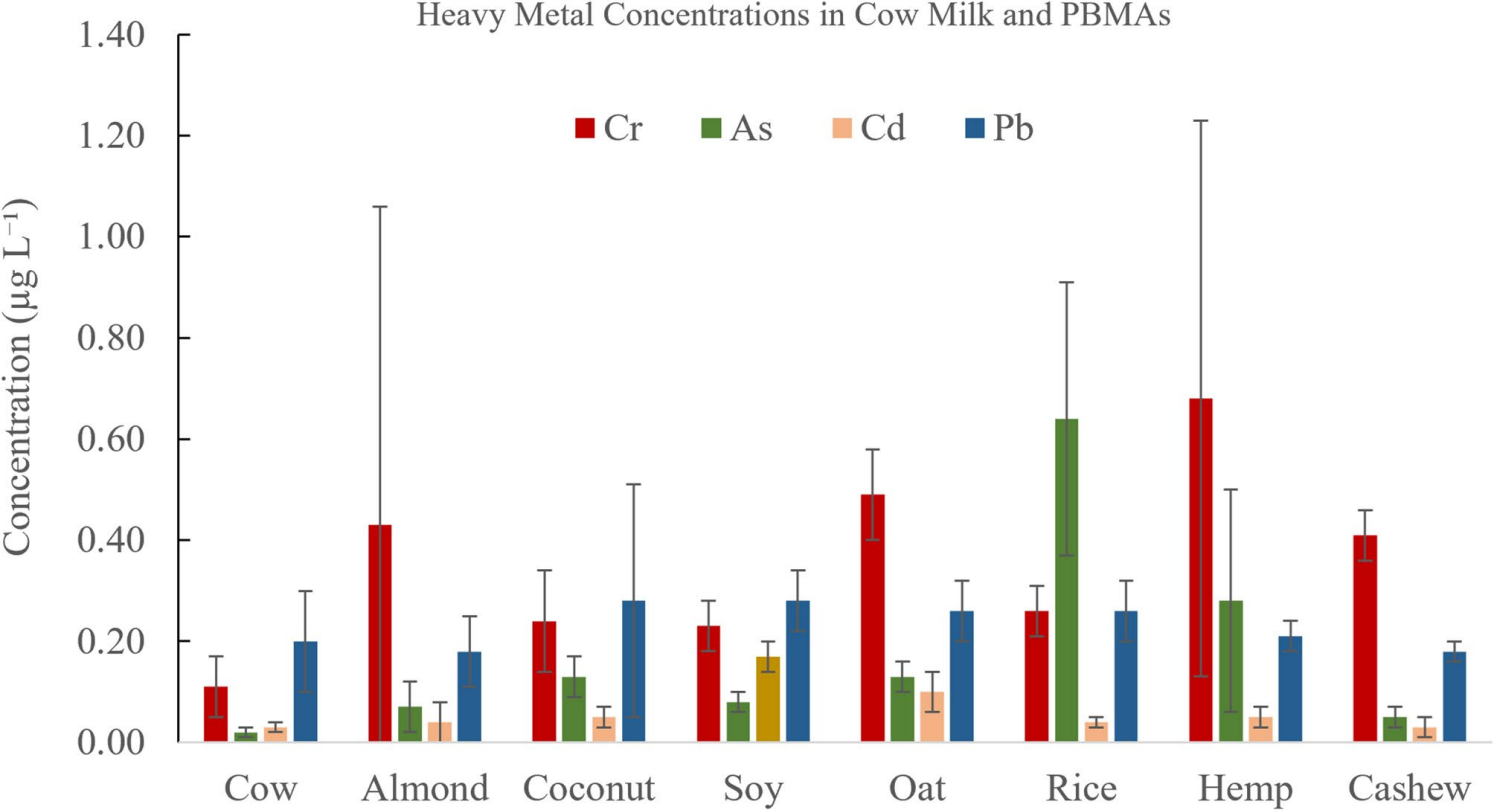
Distribution of chromium (Cr), arsenic (As), cadmium (Cd), and lead (Pb) across cow milk and major plant-based milk alternatives.

Figure 1 shows the trend of heavy metal concentration in cow and PBMAs. This figure highlights variability in heavy-metal burdens among dairy and non-dairy beverages, reflecting differences in raw materials and processing conditions. The higher concentrations of Cr, As, and Cd in PBMAs relative to cow milk observed in this study are consistent with the well-documented ability of plant matrices to accumulate trace metals from contaminated soils and irrigation water^14,47–49^. Cereals, legumes, and seeds readily take up As, Cd, and Cr, particularly when grown in regions impacted by historical agrochemical use and metal-rich groundwater. Rice is a known hyper-accumulator of inorganic arsenic, while hemp efficiently accumulates Cr and lead due to its strong phytoextraction capacity^49,50^. In contrast, cow milk typically exhibits lower metal burdens because animal feed, water, and veterinary inputs are regulated, and metals are diluted through large milk volumes^1,32^.

Although concentrations detected here fall below most regulatory thresholds, chronic dietary exposure to Pb, Cd, and Cr remains a concern, particularly for infants and children, due to neurodevelopmental, renal, and carcinogenic risks^18,51–53^.

To place these concentrations in a public health context, a conservative daily milk consumption of 250 mL was considered. At the upper range of concentrations observed in this study, As, Cd, and Cr would contribute measurable fractions of established health-based guidance values. For example, inorganic As exposure from a single serving of some plant-based milks would account for a non-trivial proportion of the World Health Organization provisional tolerable daily intake of 2.1 µg kg⁻¹ body weight day⁻¹, while Cd contributions approach levels of concern for populations with high dietary intake^13,18,48^. Although all values remained below regulatory maximum limits for conventional dairy milk, chronic low-level exposure from frequently consumed beverages represents a plausible long-term risk, particularly for children, pregnant women, and individuals who rely heavily on plant-based products^15–17,52^.

The systematically higher concentrations of several trace metals in PBMAs relative to cow milk can be explained by fundamental differences in production pathways. Cow milk reflects tightly regulated feed, water, and veterinary controls, whereas plant-based beverages directly inherit elemental signatures from soil, irrigation water, and fertilizers^1,3^. Crops such as rice, almonds, oats, and hemp are well-documented accumulators of As, Cd, and Cr due to root uptake and translocation mechanisms, particularly when grown in contaminated or historically treated agricultural soils^20,28,29,31^. Consequently, even when final beverage processing is standardized, plant-derived raw materials introduce variability and elevate trace-metal burdens in the finished products^27,32^.

### Nutrient composition

#### Macronutrients

**Fig. 2:**
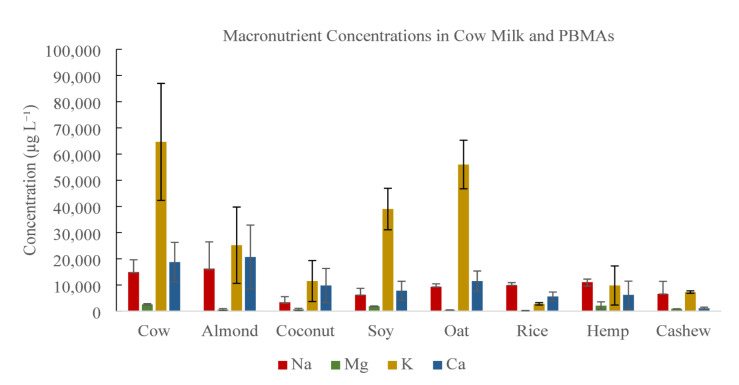
Distribution of sodium (Na), magnesium (Mg), potassium (K), and calcium (Ca) across cow milk and plant-based milk alternatives.

Figure 2 illustrates substantial variation in macronutrient composition among cow milk and PBMAs, with Ca and Na showing the greatest formulation-driven differences and K and Mg reflecting intrinsic differences between dairy and plant matrices. The observed variability in macronutrient profiles reflects both natural differences in plant raw materials and manufacturer-dependent fortification practices. Cow milk’s higher Mg and K concentrations are consistent with its natural mineral profile^1,3^. In contrast, almond and oat milks often rely on added Ca salts and mineral blends to approach dairy Ca levels^12,54,55^. The wide range observed among PBMAs highlights that nutritional equivalence to cow milk depends strongly on product formulation rather than plant source alone^1,32^.

#### Micronutrients

**Fig. 3:**
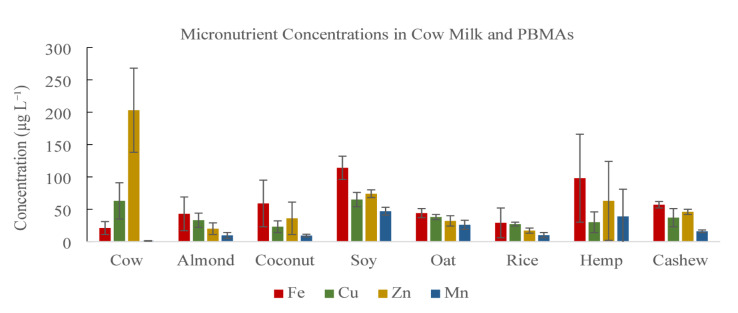
Distribution of iron (Fe), copper (Cu), zinc (Zn), and manganese (Mn) across cow milk and plant-based milk alternatives.

Figure 3 illustrates pronounced differences in micronutrient composition among cow milk and PBMAs, particularly for Fe, Zn, Cu, and Mn. PBMAs exhibited greater variability in micronutrient concentrations than cow milk, with soy, hemp, and oat milks providing substantially higher Fe and Mn levels. This pattern is consistent with compositional surveys showing that plant-based beverages can exceed dairy milk in specific trace elements, depending on raw materials and fortification, while remaining lower in others such as Zn^1,56^. Zinc is essential for immune and metabolic function^57,58^, whereas Mn, although nutritionally required, becomes neurotoxic at elevated intakes^59^. Copper supports redox-active enzymes and cellular metabolism^60^. These patterns underscore the importance of balanced micronutrient intake for consumers relying on PBMAs.

An additional complexity in interpreting mineral profiles in plant-based milks is the widespread practice of industrial fortification. Calcium, magnesium, sodium, and zinc are commonly added to PBMAs to approximate the nutritional composition of dairy milk, resulting in elevated concentrations that do not reflect intrinsic plant composition^4,54,55^. While fortification improves nutritional equivalence, it also complicates exposure assessments because added minerals coexist with naturally occurring trace contaminants derived from agricultural sources^5,6,12^. Moreover, although regulatory maximum limits exist for heavy metals in conventional milk and drinking water, no harmonized standards currently apply to plant-based milk alternatives, creating a growing regulatory gap as PBMAs become an increasingly important component of modern diets^7–9,11,61^.

### Organochlorine pesticide residues

**Fig. 4:**
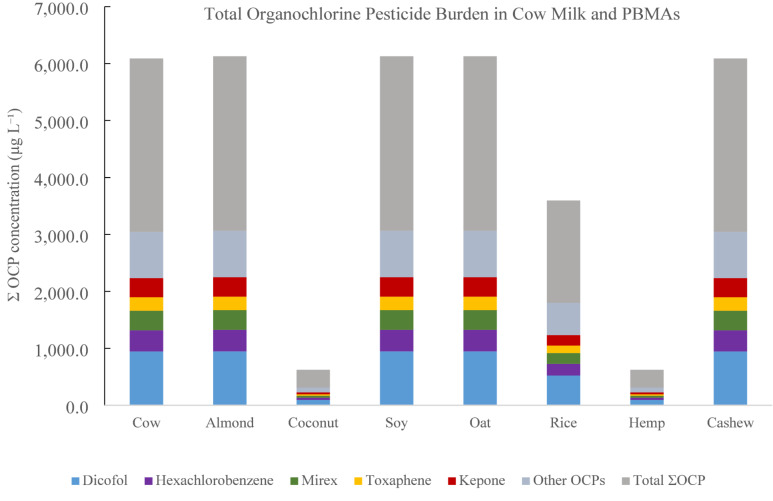
Total organochlorine pesticide burden (ΣOCP) in cow milk and plant-based milk alternatives. Bars represent the sum of mean concentrations of all 24 measured organochlorine pesticides for each milk type, with stacked segments showing the relative contributions of dicofol, hexachlorobenzene (HCB), mirex, toxaphene, and kepone, while the remaining compounds are grouped as “Other OCPs.” This representation highlights both overall pesticide load and the dominant contributors to contamination across dairy and plant-based beverages*.*

Detectable OCP residues in both dairy and PBMAs reflect the extreme environmental persistence, lipophilicity, and bioaccumulative behavior of these legacy pollutants^20,62^. As shown in Fig. 4, total OCP burden across all milk types is dominated by a small subset of legacy compounds, particularly dicofol, hexachlorobenzene, mirex, toxaphene, and kepone. For a typical 250 mL serving, this corresponds to an intake of approximately 750–770 µg of total OCPs for cow, almond, soy, and oat milks, compared with ~ 80 µg for coconut and hemp milks. Compounds such as dicofol, hexachlorobenzene, mirex, and toxaphene were widely detected across all milk categories, consistent with their long environmental half-lives and continued presence in agricultural soils, sediments, irrigation water, and atmospheric reservoirs decades after regulatory bans^61,63,64^. Because these compounds strongly partition into lipid-rich matrices, both dairy milk and plant-based beverages formulated with fats and emulsifiers serve as efficient vectors for dietary OCP exposure.

The broadly similar OCP profiles observed in cow milk and several PBMAs indicate that contamination is driven primarily by shared environmental reservoirs rather than contemporary pesticide use. Crop-derived milks inherit OCP residues from contaminated soils, groundwater, and long-range atmospheric transport^19,20,25,26,65^ whereas cow milk reflects biomagnification through animal feed, water, and lipid-rich dairy matrices^62^. The presence of high concentrations of dicofol, mirex, and hexachlorobenzene in both dairy and major PBMAs therefore underscores the continued influence of historical pesticide applications on the modern food supply.

From a toxicological perspective, the detection of multiple OCPs across all milk types is of concern because many of these compounds are endocrine-disrupting chemicals, neurotoxicants, and probable or known human carcinogens. Developmental and early-life exposure to DDT-related compounds, mirex, and hexachlorobenzene has been linked to altered neurodevelopment, thyroid and reproductive hormone disruption, immune dysfunction, and increased lifetime cancer risk^66–70^. Even low-level but continuous intake through frequently consumed beverages such as milk and PBMAs may therefore contribute meaningfully to cumulative body burdens, particularly in infants, children, and individuals who rely heavily on plant-based products.

The greater variability observed for several OCPs in rice-based beverages likely reflects heterogeneity in agricultural sourcing and environmental exposure of rice crops, which are often cultivated in flooded soils that facilitate pesticide persistence and mobilization^19,20,25,26,65^. Similarly, lower OCP concentrations in coconut and hemp milks may reflect differences in crop ecology, lipid composition, and sourcing regions, although these trends warrant further investigation with larger sample sizes and traceability data.

Importantly, unlike heavy metals and drinking-water contaminants, many OCPs lack harmonized maximum residue limits for plant-based milk alternatives, despite their rapid market growth^22–24,61^. This regulatory gap creates uncertainty in exposure assessment and risk management for consumers who substitute dairy milk with PBMAs. The widespread detection of persistent organochlorine residues in all milk categories in this study highlights the need for routine surveillance of legacy pesticides in both dairy and plant-based beverages, as well as the development of regulatory frameworks that reflect evolving dietary patterns and long-term exposure risks.

### Implications

Chronic dietary exposure to heavy metals and persistent organic pollutants disproportionately affects infants and young children due to their developing neurological and metabolic systems^52,71,72^. At the same time, adequate intake of Ca, K, and Mg is essential for cardiovascular and skeletal health^73–76^. Therefore, fortification strategies in PBMAs must balance nutritional enhancement with contaminant risk, particularly as consumers increasingly replace dairy milk with PBMAs^1,12^.

### Limitations

This study has several limitations. The sample set was relatively small and included a limited number of brands within each milk category, which may not capture the full variability of commercially available products. In addition, although samples were purchased in Houston, the products likely originated from broader national or international supply chains; therefore, the findings should not be interpreted as reflecting contamination specific to Houston or any single production region. As a cross-sectional survey, the study provides only a snapshot of products available at the time of purchase and does not account for seasonal or batch-to-batch variation. Finally, while the study quantified metals and OCP residues, it did not assess chemical speciation, bioaccessibility, or cumulative dietary exposure.

## Conclusion

This study demonstrates that both cow milk and PBMAs contain measurable levels of heavy metals, essential minerals, and legacy organochlorine pesticides. PBMAs showed greater variability in nutrient and contaminant profiles, with several products exhibiting higher concentrations of As, Cd, and Cr, reflecting uptake from plant raw materials and agricultural environments. Cow milk generally contained more consistent mineral profiles and lower trace-metal levels, but it also carried detectable pesticide residues. The widespread presence of persistent compounds such as dicofol, hexachlorobenzene, and mirex across all milk types highlights the continuing impact of historical pesticide use on the modern food supply. Although concentrations were typically below regulatory limits, chronic dietary exposure remains a concern, underscoring the need for continued monitoring and improved transparency in both dairy and plant-based products.

## Data Availability

The authors confirm that the data supporting the findings of this study are available upon reasonable request.
